# Genome-wide association study of susceptibility to hospitalised respiratory infections

**DOI:** 10.12688/wellcomeopenres.17230.2

**Published:** 2023-11-21

**Authors:** Alexander T. Williams, Nick Shrine, Hardeep Naghra-van Gijzel, Joanna C. Betts, Jing Chen, Edith M. Hessel, Catherine John, Richard Packer, Nicola F. Reeve, Astrid J. Yeo, Erik Abner, Bjørn Olav Åsvold, Juha Auvinen, Traci M. Bartz, Yuki Bradford, Ben Brumpton, Archie Campbell, Michael H. Cho, Su Chu, David R. Crosslin, QiPing Feng, Tõnu Esko, Sina A. Gharib, Caroline Hayward, Scott Hebbring, Kristian Hveem, Marjo-Riitta Järvelin, Gail P. Jarvik, Sarah H. Landis, Eric B. Larson, Jiangyuan Liu, Ruth J.F. Loos, Yuan Luo, Arden Moscati, Hana Mullerova, Bahram Namjou, David J. Porteous, Jennifer K. Quint, Regeneron Genomics Center, Marylyn D. Ritchie, Eeva Sliz, Ian B. Stanaway, Laurent Thomas, James F. Wilson, Ian P. Hall, Louise V. Wain, David Michalovich, Martin D. Tobin

**Affiliations:** 1 Department of Population Health Sciences, University of Leicester, Leicester, UK; 2 R&D, GSK, Stevenage, UK; 3 Estonian Genome Center, Institute of Genomics, University of Tartu, Tartu, Riia 23b, 51010, Estonia; 4 K.G. Jebsen Center for Genetic Epidemiology, Department of Public Health and Nursing, NTNU, Norwegian University of Science and Technology, Trondheim, Norway; 5 HUNT Research Center, Department of Public Health, Norwegian University of Science and Technology, Levanger, Norway; 6 Department of Endocrinology, Clinic of Medicine, St Olav’s Hospital, Trondheim University Hospital, Trondheim, Norway; 7 Medical Research Center Oulu, Oulu University Hospital, Center for Life Course Health Research, University of Oulu, Oulu, Finland; 8 Department of Biostatistics, University of Washington, Seattle, Washington, USA; 9 Cardiovascular Health Research Unit, Department of Medicine, University of Washington, Seattle, Washington, USA; 10 Department of Genetics and Institute for Biomedical Informatics, Perelman School of Medicine, University of Pennsylvania, Philadelphia, Pennsylvania, USA; 11 Clinic of Thoracic and Occupational Medicine, St Olav’s Hospital, Trondheim University Hospital, Trondheim, Norway; 12 Centre for Genomic and Experimental Medicine, Institute of Genetics and Cancer, University of Edinburgh, Edinburgh, UK; 13 Channing Division of Network Medicine, Brigham and Women's Hospital, Boston, Massachusetts, USA; 14 University of Washington, School of Medicine, Seattle, Washington, USA; 15 Division of Clinical Pharmacology, Department of Medicine, Vanderbilt University Medical Center, Nashville, Tennessee, USA; 16 Center for Lung Biology, Division of Pulmonary & Critical Care Medicine, Department of Medicine, University of Washington, Seattle, Washington, USA; 17 Medical Research Council Human Genetics Unit, Institute of Genetics and Cancer, University of Edinburgh, Edinburgh, UK; 18 Center for Precision Medicine Research, Marshfield Clinic Research Institute, Marshfield, Wisconsin, USA; 19 Center for Life Course Health Research, Faculty of Medicine, University of Oulu, Oulu, Finland; 20 Biocenter Oulu, University of Oulu, Oulu, Finland; 21 Unit of Primary Care, Oulu University Hospital, Oulu, Finland; 22 Department of Epidemiology and Biostatistics, School of Public Health, MRC Centre for Environment and Health, Imperial College London, London, UK; 23 Department of Life Sciences, College of Health and Life Sciences, Brunel University London, London, UK; 24 R&D, GSK, Stockley Park, UK; 25 Kaiser Permanente Washington Health Research Institute, Seattle, Washington, USA; 26 The Charles Bronfman Institute for Personalized Medicine, Icahn School of Medicine at Mount Sinai, New York, New York, USA; 27 Department of Preventive Medicine, Northwestern University, Chicago, Illinois, USA; 28 Center for Autoimmune Genomics and Etiology (CAGE), Cincinnati Children's Hospital Medical Center and University of Cincinnati College of Medicine, Cincinnati, Ohio, USA; 29 National Heart and Lung Institute, Imperial College London, London, UK; 30 Computational Medicine, Faculty of Medicine, University of Oulu, Oulu, Finland; 31 Department of Clinical and Molecular Medicine, Norwegian University of Science and Technology, Trondheim, Norway; 32 BioCore - Bioinformatics Core Facility, Norwegian University of Science and Technology, Trondheim, Norway; 33 Clinic of Laboratory Medicine, St. Olav’s Hospital, Trondheim University Hospital, Trondheim, Norway; 34 Centre for Global Health Research, Usher Institute, University of Edinburgh, Edinburgh, UK; 35 Division of Respiratory Medicine and NIHR-Nottingham Biomedical Research Centre, University of Nottingham, Nottingham, UK; 36 National Institute for Health Research, Leicester Respiratory Biomedical Research Centre, Glenfield Hospital, Leicester, UK

**Keywords:** Respiratory infections, GWAS, UK Biobank, electronic medical records

## Abstract

**Background**: Globally, respiratory infections contribute to significant morbidity and mortality. However, genetic determinants of respiratory infections are understudied and remain poorly understood.

**Methods**: We conducted a genome-wide association study in 19,459 hospitalised respiratory infection cases and 101,438 controls from UK Biobank (Stage 1). We followed-up well-imputed top signals from our Stage 1 analysis in 50,912 respiratory infection cases and 150,442 controls from 11 cohorts (Stage 2). We aggregated effect estimates across studies using inverse variance-weighted meta-analyses. Additionally, we investigated the function of the top signals in order to gain understanding of the underlying biological mechanisms.

**Results**: From our Stage 1 analysis, we report 56 signals at *P*<5×10 ^-6^, one of which was genome-wide significant ( *P*<5×10 ^-8^). The genome-wide significant signal was in an intron of *PBX3*, a gene that encodes pre-B-cell leukaemia transcription factor 3, a homeodomain-containing transcription factor. Further, the genome-wide significant signal was found to colocalise with gene-specific expression quantitative trait loci (eQTLs) affecting expression of *PBX3* in lung tissue, where the respiratory infection risk alleles were associated with decreased *PBX3* expression in lung tissue, highlighting a possible biological mechanism. Of the 56 signals, 40 were well-imputed in UK Biobank and were investigated in Stage 2. None of the 40 signals replicated, with effect estimates attenuated.

**Conclusions**: Our Stage 1 analysis implicated *PBX3* as a candidate causal gene and suggests a possible role of transcription factor binding activity in respiratory infection susceptibility. However, the *PBX3* signal, and the other well-imputed signals, did not replicate in the meta-analysis of Stages 1 and 2. Significant phenotypic heterogeneity and differences in study ascertainment may have contributed to this lack of statistical replication. Overall, our study highlighted putative associations and possible biological mechanisms that may provide insight into respiratory infection susceptibility.

## Introduction

Respiratory infections are a group of diseases characterised by infection and inflammation of the respiratory system. Respiratory infections can be grouped according to their symptomatology, anatomic involvement and causative pathogen ^1^. Upper respiratory tract infections are typically benign, self-limiting diseases, and include the common cold, pharyngitis and otitis media. However, upper respiratory tract infections can be particularly burdensome for infants and young children ^2^. Lower respiratory tract infections, on the other hand, are often life-threatening diseases that require medical intervention. In 2016, over two million deaths worldwide were caused by lower respiratory tract infections, making this group of infectious diseases the sixth leading cause of death in individuals of all ages and the leading cause of death in very young children ^3, 4^. Environmental exposures, such as indoor air pollution and inhalation of tobacco smoke, are important risk factors for upper and lower respiratory tract infections ^4^. Genetic factors may also contribute to host susceptibility to infection. Indeed, twin studies have demonstrated a genetic component in susceptibility to otitis media ^5, 6^, recurrent tonsillitis ^7^ and respiratory syncytial virus-related bronchiolitis ^8^ with heritability estimates as high as 73% ^5^. Identifying associations with genes and pathways that influence host susceptibility to infection may reveal novel therapeutic targets and opportunities for drug development.

Further to the environmental and genetic risk factors described above, primary immunodeficiencies (PIDs) are a group of disorders that affect normal immune function, often leading to increased susceptibility to infections ^9^. Activated phosphoinositide-3-kinase δ syndrome (APDS) is one such PID that is caused by gain-of-function mutations in genes encoding phosphoinositide-3-kinase δ (PI3Kδ) ^9^. In previous studies of APDS ^10, 11^, up to 96% of individuals with APDS presented with an upper respiratory tract infection, such as otitis media, and/or a lower respiratory tract infection, such as pneumonia, seemingly distinct respiratory infection diseases. These findings may motivate the need to study a broad respiratory infection phenotype—one that comprises many different kinds of respiratory infection diseases—due to the possibility of shared aetiology between distinct conditions as previously observed in the context of APDS.

In this study, we conducted a genome-wide association study (GWAS) of hospitalised respiratory infections in UK Biobank (Stage 1), utilising the Hospital Episode Statistics (HES) data. We report genetic variants that were putatively associated with hospitalised respiratory infections, of which a subset of well-imputed genetic variants was followed up in 11 independent cohorts (Stage 2). We performed an inverse variance-weighted fixed effects meta-analysis of Stages 1 and 2. Finally, we applied a range of statistical approaches in order to achieve greater insight into the biological mechanisms underlying the putative statistical associations.

## Methods

### Defining the 14 hospitalised respiratory infection phenotype

The hospitalised respiratory infection (HRI) phenotype was a composition of International Classification of Diseases, 10 ^th^ Revision (ICD-10) codes. We initially extracted all ICD-10 codes under Chapter 10: diseases of the respiratory system. Then, by manually exploring the online browser, we extracted further relevant ICD-10 codes that appear under other chapter headings that would have otherwise been missed. Following careful consideration, we restricted the ICD-10 codes to those most likely to be indicative of a respiratory infection (Table S1, *Extended data* ^12^). An ICD-10 code was deemed relevant by screening its text description, retaining those relating to clinical diagnoses and the detection of common respiratory pathogens.

### Stage 1 analysis in UK Biobank

Cases were defined by the presence of one or more of the relevant HRI ICD-10 codes (Table S1, *Extended data* ^12^) in the linked Hospital Episode Statistics (HES) data over a 20-year period—from the inception of ICD-10 coding in the UK to the end of the period covered by the version of the HES data we analysed. These data reflect all diagnoses recorded while an individual was a patient in hospital, not just the primary discharge diagnosis, and does not include outpatient hospital diagnoses. We restricted the cases to those with (1) genome-wide imputed genetic data; (2) complete information for age (at recruitment), sex and smoking status (at recruitment); (3) no 2 ^nd^ degree or closer relative (defined by a kinship estimate >0.0884 from the KING software, provided by UK Biobank) in cases only, and (4) were of European ancestry based on *k*-means clustering of the first two principal components of ancestry. Among the UK Biobank participants who were not defined as cases, i.e. individuals who had no respiratory infection codes in the secondary care data, we, separately, applied the same quality control measures as described above. Then, controls were randomly selected—to ensure computational feasibility, only a subset of controls was analysed—without replacement from the remaining individuals, using the sample function in R v3.6.1, at a ratio of five controls to every case, such that the distributions of age, sex and smoking status were broadly similar to those of the cases. Following selection of controls, the relatedness was checked between cases and controls. In 2 ^nd^ degree or closer related pairings, controls were preferentially excluded in order to maximise the number of cases in the analysis.

Genotyping was undertaken using the Affymetrix Axiom UK BiLEVE ^13^ and UK Biobank ^14^ arrays. Genotype imputation was conducted using the Haplotype Reference Consortium panel and the merged 1000 Genomes phase 3 and UK10K panels ^14^. Imputed genotypes with a minor allele count >20 (in all UK Biobank participants with genome-wide imputed genetic data) and an imputation quality score >0.5 were tested for association with the HRI phenotype.

PLINK 2.0 ^15^ was used to perform the genome-wide association study. We assessed autosomal variant associations under an additive genetic model adjusted for age (at recruitment), age ^2^, genotyping array, sex, smoking status and the first 10 principal components of ancestry. We analysed variant dosages in order to account for genotype uncertainty.

LD score regression ^16^ was used to quantify genome-wide inflation in the test statistics due to possible confounding of the genotype-phenotype associations, for example, by population stratification.

### Initial signal selection and conditional analyses

We initially defined primary signals of association according to the following criteria: minor allele frequency >0.1% (in cases and controls combined), Hardy-Weinberg exact test *P* >1×10 ^-6^ (in cases and controls combined), and an association *P* <5×10 ^-6^.

All genetic variants ±1Mb from the sentinel variant in each association signal were extracted. A conditional analysis was used to identify further, conditionally independent association signals within the 2Mb regions, using GCTA ^17, 18^. Conditionally independent signals were defined according to the same criteria as for the primary signals.

Together, the two steps outlined above describe the set of signals to be taken forward for follow-up in the 11 independent cohorts (Stage 2, described below).

### Effect of smoking behaviour

The Stage 1 analysis was adjusted for ever-smoking status. However, this may not have fully adjusted for the effect of smoking behaviour. Therefore, we assessed whether any of the association signals for HRIs were driven by smoking behaviour by testing the association between the sentinel variants from the HRI GWAS and smoking initiation (189,159 ever smokers versus 224,349 never smokers), smoking cessation (150,906 current smokers versus 45,075 ex-smokers), the number of cigarettes smoked per day (categorised, 136,391 total individuals), and heaviness of smoking index, a measure of nicotine dependence, (categorised, 31,766 total individuals). We also assessed the association with HRIs in never smokers only (8123 cases and 42,361 controls). These smoking behaviour phenotypes are discussed in more detail in the Supplementary Material ( *Extended data* ^12^). We used a *P*-value corrected for the number of sentinel variants tested to define a significant association with a smoking behaviour phenotype.

### Stage 2 cohorts

The following cohorts were included in the Stage 2 analysis: The Institute for Personalized Medicine BioMe Biobank (BioMe), Cardiovascular Health Study (CHS) ^19^, Electronic Medical Records and Genomics Network (eMERGE) ^20, 21^, Estonian Biobank ^22^, Generation Scotland: Scottish Family Health Study (GS:SFHS) ^23^, Northern Finland 1966 Birth Cohort (NFBC1966) ^24^, Orkney Complex Disease Study (ORCADES), Partners Biobank, Penn Medicine Biobank, Trøndelag Health Study (HUNT) ^25^ and Viking Health Study Shetland (VIKING). A brief summary of each of the cohorts included in the Stage 2 analysis is given in the Supplementary Material ( *Extended data* ^12^).

The Cardiovascular Health Study and Partners Biobank cohorts defined the HRI phenotype using ICD-9 codes. For this, we mapped the HRI ICD-10 codes to their ICD-9 counterparts, where possible (Table S2, *Extended data* ^12^).

### Meta-analysis of Stages 1 and 2

Of the sentinel variants in each association signal achieving *P*<5×10 ^-6^ in Stage 1, a subset was followed up in the 11 independent cohorts described above according to the following criteria: all sentinel variants with a minor allele frequency >1%, and any sentinel variant with a minor allele frequency between 0.1% and 1% that additionally had an imputation quality score >0.8. This latter criterion was used to ensure greater confidence in the genotype imputation in lower-frequency sentinel variants and, hence, in the statistical associations.

Where necessary, proxy variants, with a minimum *R ^2^* of 0.6, were substituted based on UK Biobank LD. We used the LDpair tool in the LDlink ^26^ suite of online applications to match the effect allele of proxy variants to that of the corresponding sentinel variant.

**Figure 1.**
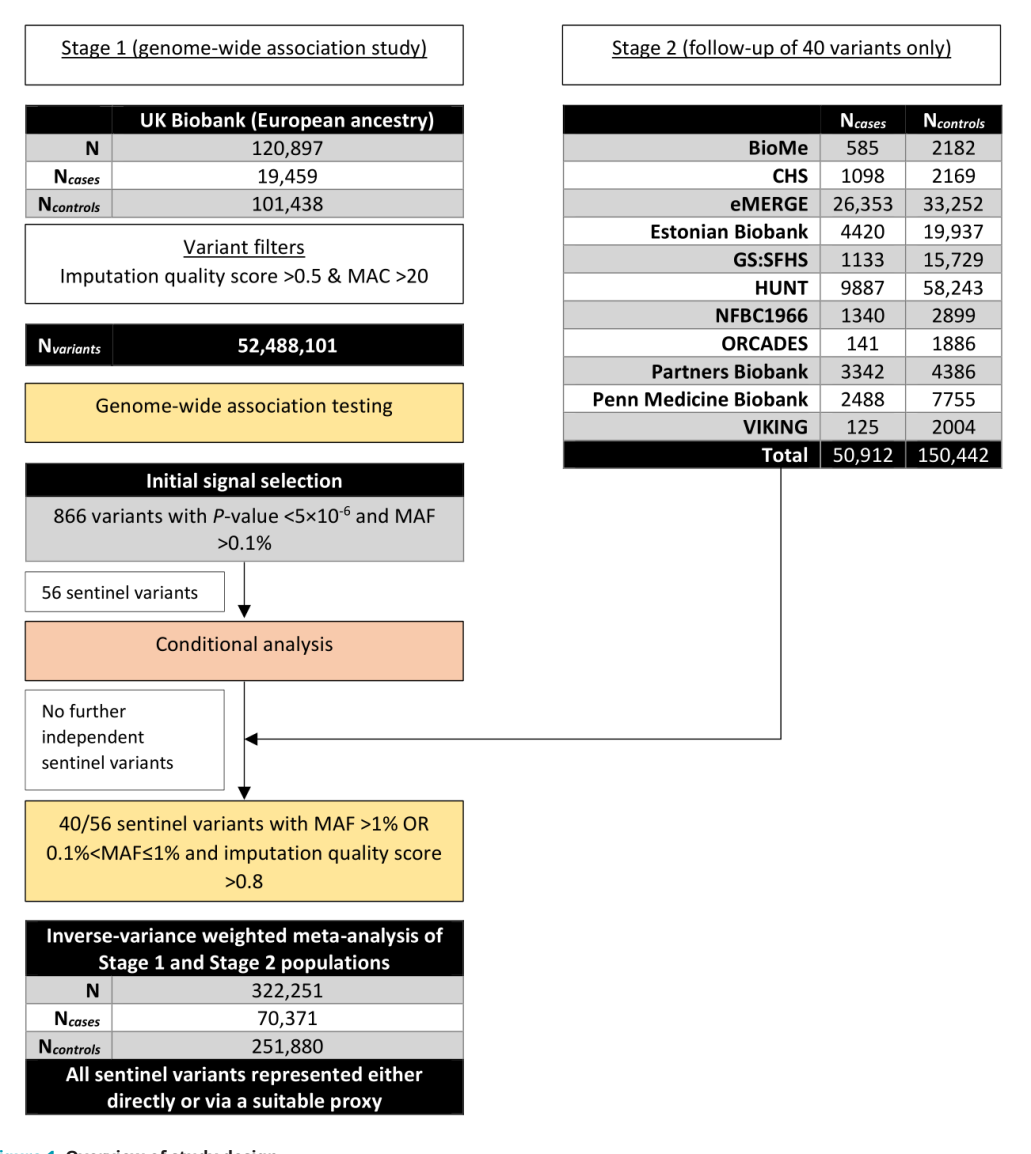
Overview of study design.

We conducted an inverse variance-weighted (IVW) fixed effects meta-analysis of association results from the Stage 2 cohorts and, separately, combined with the Stage 1 analysis using the *meta* package in R v3.6.1. We used *P*<5×10 ^-8^ in the overall meta-analysis (Stages 1 and 2) and a Bonferroni-corrected *P*-value threshold in the Stage 2 meta-analysis, corrected for the number of variants followed up, to define a replicated signal. An overview of the study design is shown in Figure 1.

### Identifying putative causal genes

***Fine-mapping.*** In order to restrict the variants in each association signal defined in the Stage 1 analysis to those most likely to be causal, we performed fine-mapping using a Bayesian method ^27^. This approach derives approximate Bayes’ factors from GWAS summary statistics, from which the posterior probability of a variant being the true causal variant (under the assumption that the true causal variant was analysed) can be calculated. The variants at each association signal can be sorted by the posterior probability and combined to create a set of variants that is 95% probable to contain the true causal variant, i.e. 95% credible set. Posterior probabilities were calculated for all variants ±1Mb from the sentinel variant in each association signal that had *R* ^2^>0.1 with the sentinel variant, using *W*=0.04 as the prior parameter, representing 95% belief that the relative risk corresponding to departure from the null model lies between 2/3 and 3/2 ^27, 28^. Association signals in the HLA region were not included in the fine-mapping.

***Functional annotation.*** To identify putative causal genes, we used the Ensembl GRCh37 Variant Effect Predictor (VEP) ^29^ to annotate all variants in the 95% credible sets. We used the following criteria to annotate variants as deleterious (all criteria implemented in VEP): labelled “deleterious” by SIFT, labelled “probably damaging” or “possibly damaging” by PolyPhen, had a CADD scaled score ≥20, labelled “likely disease causing” by REVEL, labelled “damaging” by MetaLR or “high” by MutationAssessor. The union of the variants defined by each of these methods was taken to be the set of potentially deleterious variants.

***Gene expression.*** We tested whether any variants in the 95% credible sets were associated with gene expression from three expression quantitative trait loci (eQTL) databases: 48 tissues from GTEx v7 ^30^, three major human immune cell types (CD14 ^+^ monocytes, CD16 ^+^ neutrophils, and naïve CD4 ^+^ T cells) from BLUEPRINT ^31^, and *cis*- and *trans*-eQTLs in blood from eQTLGen ^32^. A false discovery rate (FDR) of 5% was used to define a significant association with gene expression.

### Colocalisation with expression quantitative trait loci (eQTLs)

Where a variant (or variants) in the 95% credible set was found to be associated with expression of a particular gene, we assessed whether there was a shared causal variant underlying the corresponding HRI GWAS association signal and expression of the implicated gene in the highlighted tissue or cell type. We performed colocalisation using the *coloc* ^27^ package in R v3.6.1 (with default prior probabilities) and all variants within 1Mb of the sentinel variant in the corresponding HRI GWAS signal for which *P*<0.01 in either the HRI GWAS or the eQTL analysis.

In addition, we also used PICCOLO, which performs colocalisation in the absence of full summary statistics ^33^, for example if the association results for a sentinel variant only were available. In addition to eQTL data from the three eQTL databases described above, PICCOLO incorporates quantitative trait loci (QTL) data from additional sources, including protein quantitative trait loci (pQTL) data from four studies ^34– 37^. These four studies collected pQTL data for blood plasma ^34, 35^, sputum from chronic obstructive pulmonary disease (COPD) patients ^36^, and serum from asthma patients ^37^.

We used a posterior probability of >80% to identify colocalisation between the GWAS and eQTL traits for both methods described, i.e. >80% probability of a shared causal variant.

### Pathway analysis

We tested for enrichment of genes harbouring association signals in pathways defined in the MetaBase ^38^ and Gene Ontology: Biological Processes ^39, 40^ (GOBP) databases using Pascal ^41^. With Pascal, variants are mapped to genes by genomic position. To ensure computational feasibility, only GOBP pathways with >10 and <1000 genes were tested. A false discovery rate (FDR) <5% was used to define a significantly enriched pathway.

### Assessment of sentinel variants in published GWAS

We assessed whether any of the sentinel variants in the association signals were associated with other traits and diseases from existing GWAS. The traits studied included, but were not limited to, UK Biobank baseline measures (from questionnaires and physical measures), curated health outcomes from primary and/or secondary care data, and self-reported diseases and medications. *P*<5×10 ^-8^ was used to define a significant association between the sentinel variants and existing GWAS traits. Further, likely relevant, traits were also highlighted at *P*<5×10 ^-6^.

In addition, we investigated the association between the sentinel variants and four COVID-19 phenotypes from the COVID-19 Host Genetics Initiative ^42^ meta-analyses (release 6) ranging from 8779 cases (very severe COVID-19) to 112,612 cases (any COVID-19) from up to 165 cohorts worldwide. A significant association between a sentinel variant and a COVID-19 phenotype was defined using *P*<5×10 ^-8^.

### Polygenic score (PGS)

In order to assess evidence of polygenic structure in our hospitalised respiratory infection phenotype, we applied PRS-CS-auto ^43^ to create a polygenic score (PGS) using the summary statistics from a GWAS of a randomly selected half of the original Stage 1 population as the training dataset. PRS-CS-auto applies a fully Bayesian approach that automatically learns the global scaling parameter from the training dataset, and no validation dataset is needed. We tested the association of this PGS with our hospitalised respiratory infection phenotype in the half of the original Stage 1 population that was not used to generate the PGS. This association was tested using a logistic regression model adjusted for age, age2, genotyping array, sex, smoking status and the first 10 principal components of ancestry. We report the effect estimate of the PGS as a measure of polygenic structure.

### Ethics statement

UK Biobank: The human samples were sourced ethically, and their research use was in accord with the terms of the informed consents under an IRB/EC approved protocol (16/NW/0274).

Estonian Biobank: This study and the use of data acquired from biobank participants was approved by the Research Ethics Committee of the University of Tartu (Approval number 288/M-18).

Ethical approval for the GS:SFHS study was obtained from the Tayside Committee on Medical Research Ethics (on behalf of the National Health Service).

The HUNT study was approved by the Regional Committee for Medical and Health Research Ethics and written informed consent was given by all participants.

The research protocols of NFBC1966 have been approved by the Ethics Committee of the Northern Finland Ostrobothnia Hospital District and all participants have given their written informed consent.

No further ethics approvals were required for the analyses of these data.

## Results

### Defining the hospitalised respiratory infection phenotype

**Figure 2.**
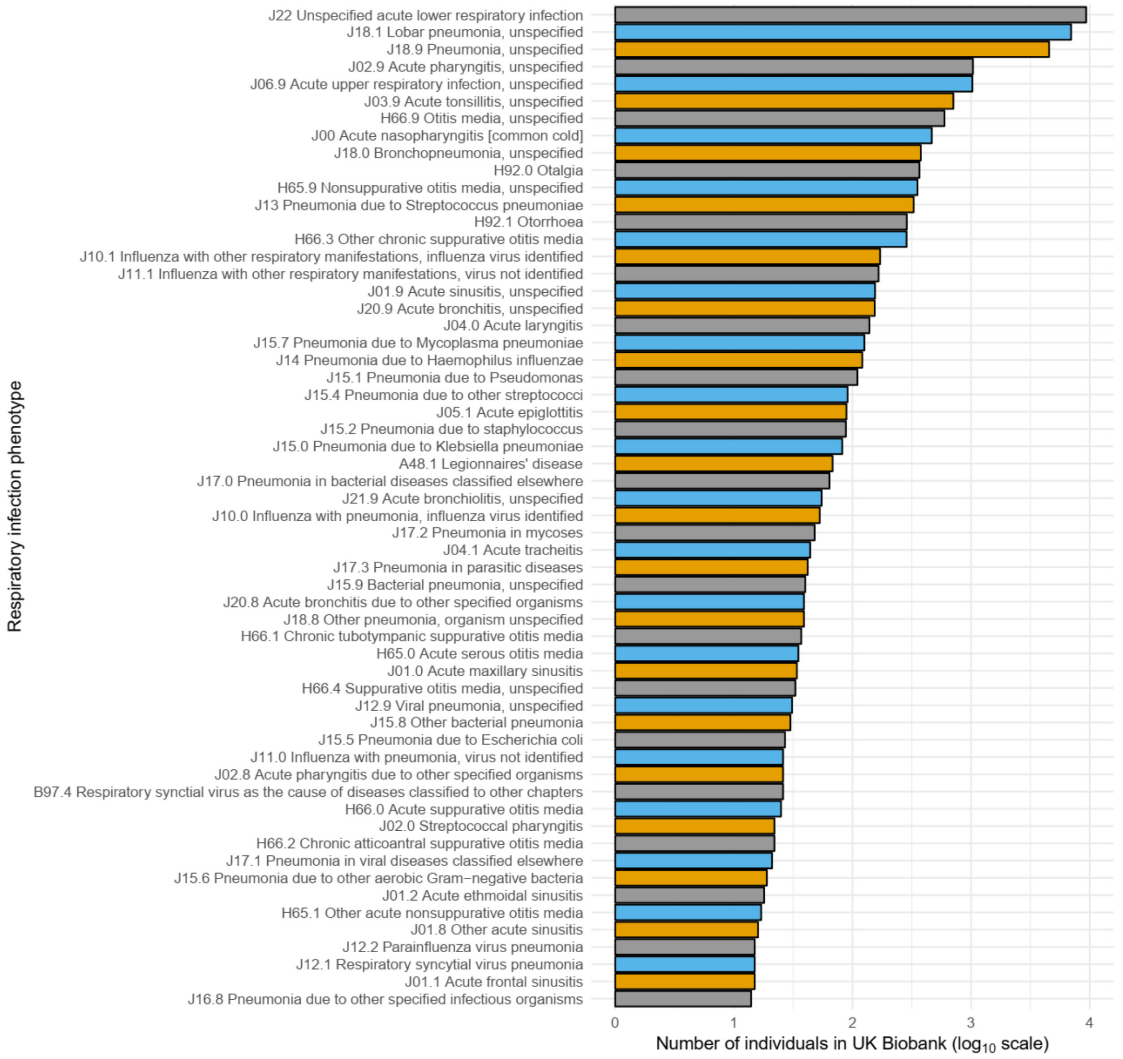
Frequency of individual ICD-10 codes used to define the 19hospitalised respiratory infection phenotype. Frequency (log _10_ scale) of individual ICD-10 codes used to define the hospitalised respiratory infection phenotype. To improve visualisation, only codes that occurred in 10 or more individuals are shown. Individuals may contribute to the overall count of more than one ICD-10 code. A description of each ICD-10 code, as well as the ICD-10 code itself, is shown.

Our hospitalised respiratory infection phenotype was a composition of 114 ICD-10 codes (Table S1, *Extended data* ^12^). Due to the specificity of certain codes (for example, “pneumonia due to *Klebsiella pneumoniae*” versus the more generic “pneumonia, unspecified”), 59 (51.8%) of these 114 ICD-10 codes occurred in fewer than 10 individuals, and 28 (24.6%) codes did not occur at all. Furthermore, 95% of cases were captured by the 16 most frequently recorded codes – the most common code, "J22 unspecified acute lower respiratory infection", accounted for more than one third (37.8%) of all cases ( Figure 2).

### Stage 1 analysis in UK Biobank

Following quality control, 19,459 cases and 101,438 controls were included in the association testing of 52,488,101 genetic variants. The intercept of LD score regression ^16^ was found to be 1.013, hence we did not correct the GWAS results for inflation ( *Methods*). The SNP heritability for the Stage 1 analysis was 9.48% (95% CI: 5.80-13.16%, liability scale). We defined 56 signals showing association at *P*<5×10 ^-6^ with hospitalised respiratory infections (HRIs), including one signal on chromosome 9 that was genome-wide significant ( *P*<5×10 ^-8^; Table S3, *Extended data* ^12^) for which the sentinel variant, rs10564495 (risk allele: A, risk allele frequency: 65.0%, risk allele count (cases): 25,806, risk allele count (controls): 131,232) was located in an intron of *PBX3*, a gene that encodes pre-B-cell leukaemia transcription factor 3, a homeodomain-containing transcription factor. The conditional analysis ^18^ did not identify further conditionally independent signals in any of the 2Mb regions.

### Effect of smoking behaviour

We assessed the association between the sentinel variants in the 56 signals and smoking behaviour traits ( *Methods* and Supplementary Material, *Extended data* ^12^). The rs10564495 variant was found to be significantly associated with smoking cessation ( *P*=1.53×10 ^-4^; Table S3, *Extended data* ^12^). The A allele for this variant was associated with 3.1% (odds ratio (OR): 0.969; 95% CI: 0.954-0.985) lower odds of quitting smoking and 7.6% (OR: 1.076; 95% CI: 1.051-1.101) greater odds of HRIs. In a stratified analysis, the association between this variant and HRIs was stronger in never-smokers than in both ever-smokers and in the overall GWAS: 8.9% (OR: 1.089; 95% CI: 1.051-1.129) greater odds of HRIs in never-smokers versus 6.6% (OR: 1.066; 95% CI: 1.034-1.099) greater odds of HRIs in ever-smokers (effect size for overall GWAS as above). These latter findings may suggest that the effect of the rs10564495 variant was not mediated by smoking behaviour.

### Meta-analysis of Stages 1 and 2

Across the 11 Stage 2 cohorts ( *Methods*), there were 50,912 additional cases and 150,442 additional controls, bringing the total number of cases to 70,371 and controls to 251,880, effectively more than tripling the number of cases included in the Stage 1 analysis ( Table 1).

**Table 1.** Summary demographics of the case-control populations in Stage 1 and each of the Stage 2 cohorts. Demographics of the case-control populations in Stage 1 and in each of the Stage 2 cohorts. *The HUNT cohort provided average year of birth rather than average age. For age, the mean and standard deviation are reported in cases and controls separately. For sex and smoking status, the number and proportion of females and never-smokers are reported in cases and controls separately.

Cohort	Sample size		Age, mean (SD)		Sex, *n* (%) – female		Smoking status, *n* (%) – never-smoker	
	Cases	Controls	Cases	Controls	Cases	Controls	Cases	Controls
**UK Biobank** **(discovery)**	19,459	101,438	59.1 (7.7)	59.0 (7.7)	9280 (47.7)	48,312 (47.6)	8123 (41.7)	42,361 (41.8)
**BioMe**	585	2182	60.0 (17.2)	56.9 (17.4)	335 (57.3)	1058 (48.5)	328 (56.1)	1205 (55.2)
**CHS**	1098	2169	72.5 (5.1)	72.3 (5.5)	647 (58.9)	1342 (61.9)	476 (43.4)	1088 (50.2)
**eMERGE**	26,353	33,252	67.3 (24.0)	58.6 (24.8)	11,644 (44.2)	16,964 (51.0)	18,265 (69.3)	26,938 (81.0)
**Estonian Biobank**	4420	19,937	59.2 (17.9)	58.9 (17.6)	2961 (67.0)	13,230 (66.4)	2446 (55.3)	10,916 (54.8)
**GS:SFHS**	1133	15,729	41.8 (17.3)	47.4 (14.4)	651 (57.5)	9223 (58.6)	535 (47.2)	8271 (52.6)
**HUNT** *	9887	58,243	1940 (16.8)	1950 (17.7)	4794 (48.5)	31,203 (53.6)	3106 (31.4)	25,153 (43.2)
**NFBC1966**	1340	2899	31.1 (0.4)	31.1 (0.4)	534 (39.9)	1795 (61.9)	820 (61.2)	1628 (56.2)
**ORCADES**	141	1886	55.6 (19.5)	53.6 (15.0)	93 (66.0)	1131 (60.0)	86 (61.0)	1159 (61.5)
**Partners Biobank**	3342	4386	62.5 (15.5)	59.0 (16.6)	1959 (58.6)	2387 (54.4)	2023 (60.5)	2797 (63.8)
**Penn Medicine** **Biobank**	2488	7755	69.7 (13.6)	70.5 (13.6)	916 (36.8)	2569 (33.1)	953 (38.3)	3398 (43.8)
**VIKING**	125	2004	45.4 (16.8)	50.1 (15.1)	71 (56.8)	1208 (60.3)	72 (57.6)	1100 (54.9)
**Total**	70,371	251,880			33,885 (48.2)	130,422 (51.8)	37,233 (52.9)	126,014 (50.0)

**Figure 3.**
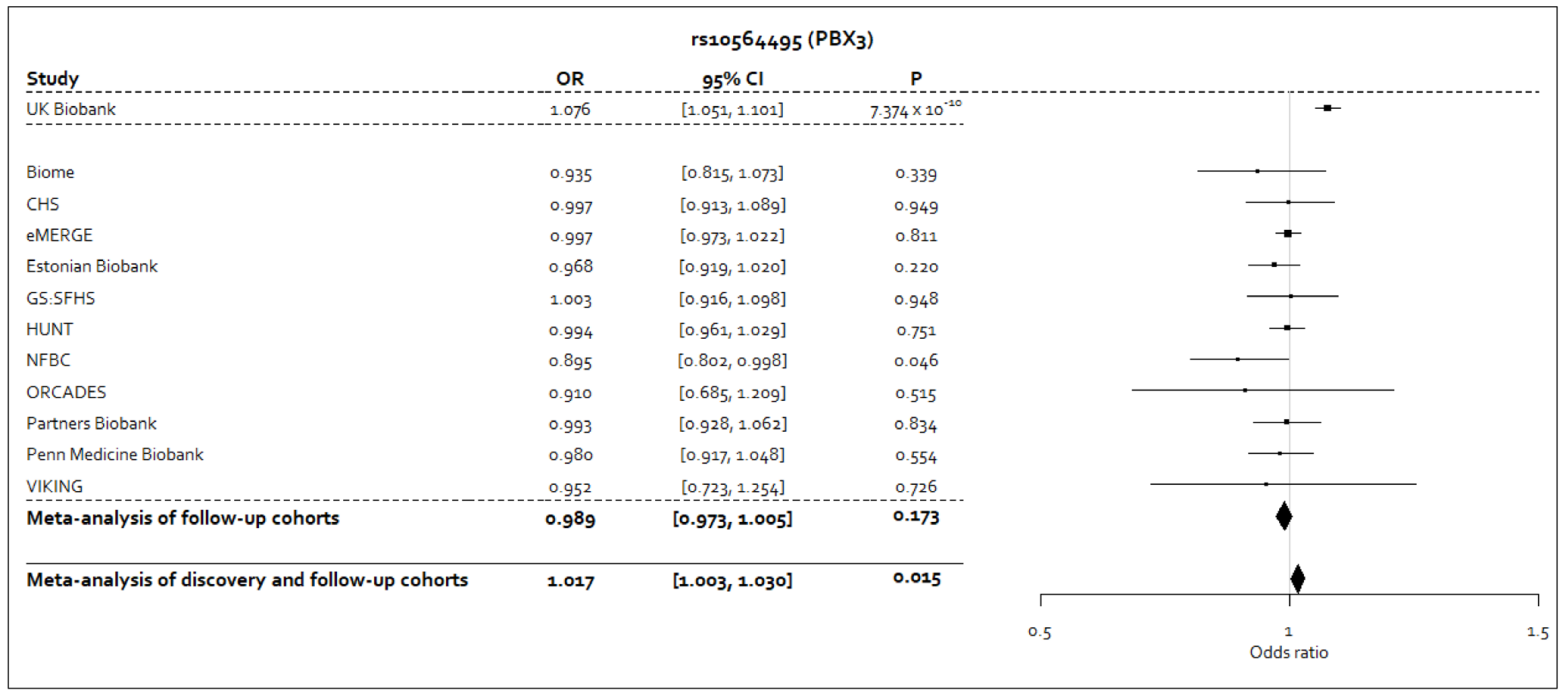
Forest plot for the sentinel variant in the genome-wide significant signal from the Stage 1 analysis following meta-analysis of Stages 1 and 2. Forest plot for the sentinel variant, rs10564495, in the genome-wide significant signal identified in the Stage 1 following inverse variance-weighted fixed effects meta-analysis of results from Stages 1 and 2. The A allele for this variant was taken to be the coded allele. Where a proxy variant was used, which was consistently the rs10819083 variant, the T allele was taken to be the allele that corresponds to the A allele of the rs10564495 variant, as reported by the LDpair tool in the LDlink ^26^ suite of online applications.

In Stage 2, we followed up a total of 40 variants. The availability of each variant across the 11 Stage 2 cohorts is shown in Table S4, *Extended data* ^12^. In the meta-analysis of Stages 1 and 2, no variants achieved *P*<5×10 ^-8^ (Table S5, *Extended data* ^12^). Furthermore, in the Stage 2 meta-analysis, no variants met a Bonferroni-corrected *P*-value threshold for 40 tests ( *P*<0.05/40=1.25×10 ^-3^). The effect estimates in the Stage 2 cohorts for rs10564495-A, or its proxy rs10819083-T, were consistently in the opposite direction, or were close to the null value, to the effect estimate from the Stage 1 analysis ( Figure 3). In the meta-analysis of Stages 1 and 2 for the rs10564495 variant, we observed an *I ^2^* statistic of 70.8% (95% CI: 45.9%-84.2%; *P*=0.0002), representing significant heterogeneity in the meta-analysis for this variant.

### Identifying putative causal genes

***Fine-mapping.*** There were 107 variants in the 95% credible set at the genome-wide significant locus from the Stage 1 analysis. The sentinel variant, rs10564495, at this locus was assigned 16.2% probability of being causal, the highest probability in the corresponding 95% credible set (Table S6, *Extended data* ^12^).

***Functional annotation.*** According to the criteria defined in *Methods*, there were six variants in five unique genes across four signals that were annotated as deleterious (Table S7, *Extended data* ^12^): *DNAH6* (rs72832548 and rs72836490), *ZNF608* (rs10040793), *PBX3* (rs7849076 and rs1411352), *RNU6-457P* (rs2172310) and *RBFOX1* (rs2172310). The two missense variants in *DNAH6* (rs72832548 and rs72836490) were low frequency (minor allele frequencies of 0.55% and 0.56%, respectively) and result in amino acid changes from serine to glycine and alanine to threonine, respectively. The consequence(s) of these base changes has not been reported. *DNAH6* encodes a protein that is involved in regulating motile ciliary beating ^44, 45^ and has been implicated in primary ciliary dyskinesia ^46^, a disorder characterised by chronic respiratory tract infections. *PBX3* houses the genome-wide significant signal from the Stage 1 analysis. However, the two variants in *PBX3* annotated as deleterious were non-coding (Table S7, *Extended data* ^12^).

### Gene expression and colocalisation with expression quantitative trait loci (eQTLs)

**Figure 4.**
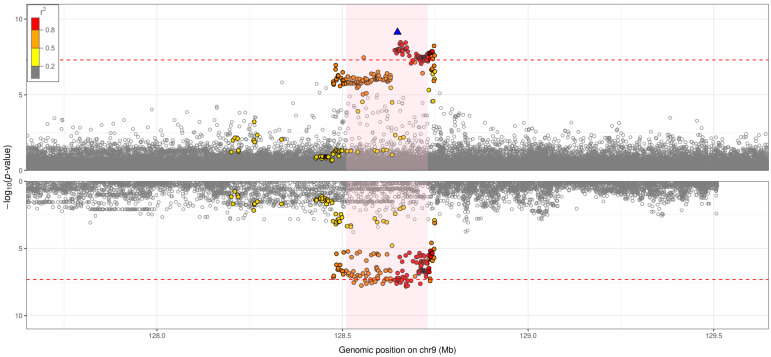
Hospitalised respiratory infection GWAS versus eQTL for *PBX3* in lung tissue (GTEx v7): probability of colocalisation = 87%. Each point corresponds to a genetic variant, with genomic position (GRCh37) on the *x*-axis and –log _10_( *p*-value) on the *y*-axis. The top plot shows regional association results for the genome-wide significant signal (sentinel variant: rs10564495) from the hospitalised respiratory infection GWAS. The bottom plot shows regional association results for the genome-wide significant signal from the eQTL analysis. The plotting window extends 1Mb either side of the sentinel variant in the region. The sentinel variant is represented by a blue triangle, with all other genetic variants in the region coloured according to the extent of pairwise linkage disequilibrium with the sentinel variant: red points reflect genetic variants that have *r ^2^* >0.8 with the sentinel variant, orange points reflect genetic variants that have 0.5< *r ^2^* ≤0.8 with the sentinel variant, yellow points reflect genetic variants that have 0.2< *r ^2^* ≤0.5 with the sentinel variant, and grey points reflect genetic variants that have *r ^2^* ≤0.2 with the sentinel variant. The area shaded in light pink represents the gene implicated by the eQTL analysis. The red dashed line represents a *p*-value threshold of 5×10 ^-8.^

Using GTEx v7 ^30^ data, the genome-wide significant signal from the Stage 1 analysis was found to colocalise (PP>80%) with *PBX3*-specific eQTLs in heart atrial appendage tissue, tibial artery tissue, not-sun-exposed suprapubic skin tissue, stomach tissue, lung tissue, aortic artery tissue, and sigmoid colon tissue ( Figure 4 and Supplementary Figures, *Extended data* ^12^). The HRI risk alleles were consistently associated with decreased *PBX3*-specific gene expression in all of the aforementioned tissues (Table S8, *Extended data* ^12^). We also found colocalisation between the genome-wide significant signal and expression of the proximal *GOLGA1* gene in sun-exposed lower leg skin tissue (PP=81%). We did not identify additional colocalisation using BLUEPRINT ^31^ or eQTLGen ^32^ data.

Using PICCOLO ^33^, the genome-wide significant signal from the Stage 1 analysis was found to additionally colocalise (PP>80%) with *PBX3*-specific eQTLs in CD4/8 ^+^ naïve T cells, coronary artery tissue and whole blood (Table S11, *Extended data* ^12^). PICCOLO did not highlight colocalisation between eQTLs and any proximal genes to *PBX3*. At the time of analysis, PICCOLO did not provide effect estimates for the eQTL traits. Therefore, we queried the Open Targets Genetics ^47^ portal in order to assess directionality for these additional eQTL traits. The HRI risk alleles were associated with decreased *PBX3*-specific expression in coronary artery tissue. Summary statistics for the T cell and whole blood traits were not available, however.

For the remaining signals, the chromosome 5 signal (sentinel variant: rs7730012) was found to colocalise with *ZNF608*-specific eQTLs in tibial artery tissue (GTEx v7 ^30^) using the *coloc* ^27^ method (Supplementary Figure 1, *Extended data* ^12^). Additional results from PICCOLO ^33^ can be seen in Table S11 ( *Extended data* ^12^).

### Pathway analysis

We tested for significant enrichment of genes from the HRI GWAS in known pathways: 1383 pathways from the MetaBase ^38^ resource and 6405 pathways from the Gene Ontology: Biological Processes ^39, 40^ resource ( *Methods*). We did not identify any significantly enriched pathways at a false discovery rate of 5%.

### Assessment of sentinel variants in published GWAS

The A allele of the rs10564495 variant was associated with increased overall health rating, increased odds of requiring the use of dentures and decreased standing height at *P* <5×10 ^-8^ (Table S12, *Extended data* ^12^), and decreased lung function and increased odds of various respiratory disease phenotypes, including respiratory infections, at *P* <5×10 ^-6^ (Table S13, *Extended data* ^12^). Significant associations for the other sentinel variants are presented in Tables S12&S13 and include various respiratory infection phenotypes such as acute pharyngitis and pneumonia.

Finally, there were no significant associations found between any of the sentinel variants and the four COVID-19 phenotypes from the COVID-19 Host Genetics Initiative ^42 ^(Table S14, *Extended data* ^12^).

### Polygenic score (PGS)

There were 9730 cases and 50,719 controls randomly selected for the GWAS from which the PGS was constructed. When we tested the association between the PGS and our hospitalised respiratory infection phenotype in the other half of the Stage 1 population (9729 hospitalised respiratory infection cases and 50,719 controls), we found an 8.1% (95% CI: 5.8%-10.5%; *P*=2.50×10 ^-12^) increase in disease odds per standard deviation unit increase in the PGS.

## Discussion

We conducted one of the largest GWAS of respiratory infections to date, combining data from UK Biobank and 11 international cohorts.

In our Stage 1 analysis, the strongest association signal was in an intron of the *PBX3* gene, which encodes the pre-B-cell transcription factor 3 protein. *PBX3* contributes to DNA-binding transcription factor activity and sequence-specific DNA binding. The hospitalised respiratory infection risk alleles at this locus were associated with decreased expression of *PBX3* in lung tissue (Table S8, *Extended data* ^12^). In a recent preprint, *PBX3* was found to be associated with pneumonia in almost 25,000 cases from UK Biobank and FinnGen ^48^. The authors also found that genetic variants in *PBX3* were associated with *PBX3* expression in lung tissue (effect direction not reported). In a study of a range of infectious diseases using 23andMe data, including some respiratory infections such as pneumonia and childhood ear infections, neither *PBX3*, nor any neighbouring genes, were found to be associated with the diseases studied ^49^. However, it should be noted that the respiratory infection phenotypes in the 23andMe study were defined from self-reported questionnaire data which may have been subject to recall bias, particularly for diseases that occurred during childhood.

Evidence that *PBX3* is a functionally significant transcription factor in a range of cancers, in addition to its expression being linked to more aggressive disease and shorter overall survival, has been reported ^50^. Cancer patients are more susceptible to infections for a number of reasons. One such reason may be due to the receipt of immunosuppressants compromising the individual’s immune system, resulting in greater risk of opportunistic infection, as has been observed in lung cancer patients ^51^.

We followed up 40 signals from our Stage 1 analysis in 11 independent cohorts (Stage 2). None of the 40 signals surpassed *P*<5×10 ^-8^ in the meta-analysis of Stages 1 and 2, highlighting the importance of statistical replication and the potential influence of winner’s curse bias in our Stage 1 analysis. However, it is possible that there was significant phenotypic heterogeneity, reflected in a significant *I* ^2^ statistic for many of the 40 signals (Table S5, *Extended data ^12^*), between cohorts owing to differences in exposure to circulating pathogens, health care systems and coding practices which may have influenced the representation of particular infections in the medical record data. For example, the respiratory infection phenotype in UK Biobank was defined using ICD-10 codes (Table S1, *Extended data* ^12^). In two of the Stage 2 cohorts, the corresponding phenotype was defined entirely or partly by ICD-9 codes (Table S2, *Extended data* ^12^). As there is no exact mapping between ICD-9 and ICD-10 codes, the resulting phenotypes may differ and give rise to greater heterogeneity between cohorts. In addition, controls were not selected for similar distributions of age, sex and smoking status in the Stage 2 cohorts which, in some cases, led to large differences in the distributions of the aforementioned factors between cases and controls for some cohorts ( Table 1).

In addition to considerations around phenotypic differences, it is important to consider that the association test statistics for specific variants may be subject to ascertainment biases given that not all variants were available to study in all cohorts and participant ascertainment strategies – such as hospital-based versus population-based recruitment - varied between studies. 

We applied a range of statistical techniques to further understand the biological mechanisms underlying the statistical associations identified in Stage 1. Following fine-mapping ^28^, the sentinel variant, rs10564495, in *PBX3* was attributed 16.2% probability of being causal for the GWAS trait among a set of 107 genetic variants that was attributed 95% probability of containing the true causal variant. These 107 genetic variants were located in, or slightly upstream of, *PBX3*. We found two variants in *PBX3* that were annotated as deleterious. However, these variants were non-coding/regulatory region variants (Table S7, *Extended data* ^12^), which may suggest that these variants are involved in gene expression. Further work is needed to understand the role of these particular variants in influencing susceptibility to hospitalised respiratory infections. In a colocalisation analysis, the *PBX3* signal was found to colocalise with *PBX3*-specific eQTLs in a range of tissues and cell types including, but not limited to, lung tissue, CD4/8 ^+^ T cells and whole blood. At the *PBX3* locus, the alleles that were associated with increased risk of hospitalised respiratory infections were also associated with decreased expression of *PBX3* in the tissues and cell types highlighted and may implicate *PBX3* as a candidate causal gene. Furthermore, we found that the *PBX3* sentinel variant was associated with overall health rating, denture use and standing height at *P*<5×10 ^-8^, and a broader respiratory system disease phenotype and FEV _1_ at *P*<5×10 ^-6^ when looking across a large number of published GWAS. These associations, particularly those with FEV _1_ and standing height, may implicate lung function as a driver of the *PBX3* association signal. In the absence of independent replication, however, any interpretation of functional evidence relating to the *PBX3* signal, or the remaining signals, must be viewed with caution. We presented findings for additional signals from our Stage 1 analysis. However, since these signals were not genome-wide significant in Stage 1, or in the meta-analysis of Stages 1 and 2, our interpretation of the functional evidence relating to these signals should be viewed with caution.

As with all research based on medical records, misclassification of diagnoses may have occurred, and we did not have the benefit of microbiological or virological data to confirm the infective agent. Nevertheless, the use of medical records enabled us to study much larger sample sizes than have been attained in studies that do not use such data—historically, GWAS that define cases of respiratory infection by other means included fewer than 1000 cases ^52– 57^. We combined multiple respiratory infection codes to define our overall phenotype, motivated by previous findings in the context of APDS, which resulted in a larger sample size but likely increased heterogeneity. Controls were individuals with no evidence of having had a respiratory infection in hospital, but we did not consider other data sources, such as primary care data, where there may be records of respiratory infection among the controls, reflecting misclassification and a possible loss of statistical power. Gene expression data generated from healthy tissues and cells, as is the case for the three eQTL datasets we used, may not accurately represent the biological landscape during disease. Furthermore, the gene expression data provides insight into gene expression at the tissue level, but some effects may be mediated via specific cell types within a tissue that may have been missed in our analysis. Finally, we restricted our analysis to unrelated individuals of European ancestry in order to limit the potential impact of population stratification and cryptic relatedness. However, this may limit the generalisability of the results we report.

Using genome-wide SNPs, we observed a highly-significant SNP heritability, with a point estimate of 9.48% (liability scale), which is higher than estimates provided in previous large GWAS of respiratory infections ^49, 50^. Given this SNP heritability, the lack of signals reaching genome-wide significance in the overall (Stage 1+2) meta-analysis is consistent with a polygenic trait – that is, many variants of individually small effect. In our polygenic score analysis, we observed an 8.1% (95% CI: 5.8%-10.5%) increase in hospitalised respiratory infection odds per standard deviation unit increase in the polygenic score, which is also consistent with polygenic architecture of hospitalised respiratory infections. Despite this being the largest such study undertaken to date, these findings would suggest that discovery of additional genetic associations with hospitalised respiratory infections would require even larger sample sizes, and ideally discovery and follow-up populations subject to relatively homogeneous approaches to coding respiratory infections.

To conclude, genetic variants in *PBX3* were found to be associated with hospitalised respiratory infection susceptibility in UK Biobank, which may implicate transcription factor binding activity in susceptibility to a general respiratory infection phenotype. However, this finding did not replicate in independent cohorts. Future genome-wide association studies of hospitalised respiratory infection susceptibility would benefit from larger sample sizes and reduced phenotypic heterogeneity that may arise when utilising linked electronic healthcare records across different healthcare systems.

## Data Availability

This research has been conducted using the UK Biobank resource under applications 648 and 4892. The genetic and phenotypic UK Biobank data can be requested upon application to the UK Biobank resource for all bona fide researchers (see https://www.ukbiobank.ac.uk/researchers/ for more details). Figshare: WilliamsAT_prePMID_HRI.tsv.gz. https://doi.org/10.6084/m9.figshare.16622062 ^58^. Figshare: Williams_et_al_extended_data. https://doi.org/10.6084/m9.figshare.16622191 ^12^. This project contains the following extended data: supplementary_material.docx (Supplementary material and methods) supplementary_figures.docx (Supplementary figures) tableS1_icd10_codes.csv (Table S1, ICD-10 codes used to define the hospitalised respiratory infection phenotype). tableS2_icd9_codes.txt (Table S2, ICD-9 codes used to define the hospitalised respiratory infection phenotype in CHS and Partners Biobank). tableS3_discovery_summstats_sentinels.csv (Table S3, Summary statistics for the 56 sentinel variants from the discovery GWAS in UK Biobank). tableS4_followup_availability_sentinels.csv (Table S4, Availability of the 40 sentinel variants in the 11 follow-up cohorts). tableS5_metaanalysis_sentinels.csv (Table S5, Results of the inverse variance-weighted fixed effects meta-analysis). tableS6_finemapping_results.csv (Table S6, Fine-mapping of the 56 association signals). tableS7_annotation_results.csv (Table S7, Functional annotation of variants in the 95% credible sets). tableS8_geneexpression_results_gtex.csv (Table S8, Association between variants in the 95% credible sets and gene expression across 48 tissues from GTEx v7). tableS9_geneexpression_results_blueprint.csv (Table S9, Association between variants in the 95% credible sets and gene expression across three major human immune cell types from BLUEPRINT). tableS10_geneexpression_results_eqtlgen.csv (Table S10, Association between variants in the 95% credible sets and gene expression (cis-eQTLs) from eQTLGen). tableS11_coloc_piccolo_results.csv (Table S11, Colocalisation results from PICCOLO). tableS12_gwsig_lookup_results.csv (Table S12, Look-up of the sentinel variants in the 56 association signals in existing GWAS). tableS13_suggestive_lookup_results.csv (Table S13, Look-up of the sentinel variants in the 56 association signals in existing GWAS). tableS14_covid19hgi_lookup_results.csv (Table S14, Look-up of the sentinel variants in the 56 association signals in the COVID-19 Host Genetics Initiative meta-analysis results). Data are available under the terms of the Creative Commons Attribution 4.0 International license (CC-BY 4.0).
