# Supplementary material for: Genome-wide association study of susceptibility to hospitalised respiratory infections
Source: Wellcome Open Res. 2023 Nov 21;6:290. Originally published 2021 Oct 27. [Version 2] doi: 10.12688/wellcomeopenres.17230.2 (PMC11362726; doi:10.12688/wellcomeopenres.17230.2)
Supplement: Peer review history

# Reviewer report: Reviewer response for version 2

**DOI:** 10.21956/wellcomeopenres.21409.r70307

**Published:** 2024-08-29

**Authors:** Chikashi Terao

**Affiliations:** 1 Laboratory for Statistical and Translational Genetics, RIKEN Center for Integrative Medical Sciences, Yokohama, Japan

I have no further comments.

Is the work clearly and accurately presented and does it cite the current literature?

Partly

If applicable, is the statistical analysis and its interpretation appropriate?

Partly

Are all the source data underlying the results available to ensure full reproducibility?

Partly

Is the study design appropriate and is the work technically sound?

Partly

Are the conclusions drawn adequately supported by the results?

No

Are sufficient details of methods and analysis provided to allow replication by others?

Partly

Reviewer Expertise:

Genetics

I confirm that I have read this submission and believe that I have an appropriate level of expertise to confirm that it is of an acceptable scientific standard.

# Reviewer report: Reviewer response for version 2

**DOI:** 10.21956/wellcomeopenres.21409.r76720

**Published:** 2024-03-22

**Authors:** Shizheng Qiu

**Affiliations:** 1 Harbin Institute of Technology, Harbin, China

With the COVID-19 pandemic, there is increasing concern about the hazards posed by respiratory infections and related diseases. This study is contextualized within this era and contributes to the community. The authors conducted a genome-wide association study on 19,459 hospitalized cases of respiratory infections and 101,438 controls from the UK Biobank, identifying a significant genetic locus located at PBX3. Unfortunately, this locus was not successfully replicated in the replication cohort. It is worth mentioning that the heritability of respiratory infections is close to 10%, which exceeds that of COVID-19 by far. A major limitation of this study is that the authors defined participants with one or more respiratory infections as cases, with a considerable portion of cases having unclear specific diseases. Authors also faced difficulty in unifying the phenotype definitions between the discovery and replication cohorts. It is evident that conducting GWAS for each respiratory infection caused by different bacteria or viruses individually would be challenging due to insufficient samples or statistical significance, so the authors need not make modifications. Furthermore, exploring the potential risk factors for respiratory infections would be a significant contribution to the community.

Is the work clearly and accurately presented and does it cite the current literature?

Yes

If applicable, is the statistical analysis and its interpretation appropriate?

Yes

Are all the source data underlying the results available to ensure full reproducibility?

Yes

Is the study design appropriate and is the work technically sound?

Yes

Are the conclusions drawn adequately supported by the results?

Yes

Are sufficient details of methods and analysis provided to allow replication by others?

Yes

Reviewer Expertise:

GWAS

I confirm that I have read this submission and believe that I have an appropriate level of expertise to confirm that it is of an acceptable scientific standard, however I have significant reservations, as outlined above.

# Reviewer report: Reviewer response for version 1

**DOI:** 10.21956/wellcomeopenres.19042.r49741

**Published:** 2022-04-27

**Authors:** Gregory Joseph Fonseca

**Affiliations:** 1 Department of Medicine, Division of Quantitative Life Sciences, Meakins-Christie Laboratories, McGill University Health Centre, Montreal, QC, Canada

In this study, the authors curate respiratory infections from the UK Biobank and compare genetic variation with controls. The authors find 40 statistically significant variants. The authors then provide functional annotation and predict cell type expression.

- Are the controls patients that have not had a hospitalized respiratory infection? This was unclear to me.
- Is there a control for age bias? Younger patients would have less chance of contracting a respiratory infection.
- Gene expression in healthy tissues may not be a good measure of gene expression during disease. Also, this is missing the resident cells that would direct the initial response.
- If the data is regressed against the smoking state, do the results hold, such as with rs10564495?
- Do the follow-up cohorts represent a distinctly different population? Is this the reason no variants were significant?
- Did the variants not appear in any of the 11 follow-up cohorts? It may be worth testing these systematically in case a few of your cohorts are hiding any results due to an inherent bias in the cohort.
- How many cases were found with variants in PBX3?
- If the PBX3 variants are non-coding, what is the proposed effect? Are they splicing, are they in enhancer-like elements? Are they in non-coding RNA-like elements?

Is the work clearly and accurately presented and does it cite the current literature?

Yes

If applicable, is the statistical analysis and its interpretation appropriate?

Partly

Are all the source data underlying the results available to ensure full reproducibility?

Yes

Is the study design appropriate and is the work technically sound?

Yes

Are the conclusions drawn adequately supported by the results?

Partly

Are sufficient details of methods and analysis provided to allow replication by others?

Partly

Reviewer Expertise:

Bioinformatics, genomics, viral infection

I confirm that I have read this submission and believe that I have an appropriate level of expertise to confirm that it is of an acceptable scientific standard, however I have significant reservations, as outlined above.

# Response

**Published:** 2023-03-29

**Authors:** Alexander Williams

**Affiliations:** University of Leicester, UK

*In this study, the authors curate respiratory infections from the UK Biobank and compare genetic variation with controls. The authors find 40 statistically significant variants. The authors then provide functional annotation and predict cell type expression.*

- *Are the controls patients that have not had a hospitalized respiratory infection? This was unclear to me.*

Thank you for highlighting that our definition of controls could be made clearer. We have added a sentence to "Stage 1 analysis in UK Biobank" (Methods) to clarify this.

- *Is there a control for age bias? Younger patients would have less chance of contracting a respiratory infection.*

Our GWAS model was adjusted for age and age ^2^. However, age is not expected to confound a GWAS association as genotypes are randomly assigned at conception.

- *Gene expression in healthy tissues may not be a good measure of gene expression during disease. Also, this is missing the resident cells that would direct the initial response.*

We agree with your comment and we have added a sentence to the Discussion (paragraph 7) to reflect this.

- *If the data is regressed against the smoking state, do the results hold, such as with rs10564495?*

Our GWAS model was adjusted for ever smoking status. In a smoking-stratified analysis, we saw no difference in the effect estimate for rs10564495 in ever smokers compared to never smokers (Table S3, *Extended data*).

- *Do the follow-up cohorts represent a distinctly different population? Is this the reason no variants were significant?*

We have added further text to the Discussion to present several hypotheses for the lack of replication.

- *Did the variants not appear in any of the 11 follow-up cohorts? It may be worth testing these systematically in case a few of your cohorts are hiding any results due to an inherent bias in the cohort.*

Thank you for highlighting that this aspect of the analysis could have been made clearer. This information was provided in Table S4 (Extended data); however we have added text to "Meta-analysis of Stage 1 and Stage 2" (Results) to aid interpretation.

- *How many cases were found with variants in PBX3?*

We have added a sentence that describes the risk allele frequency for the *PBX3* sentinel variant separately in cases and controls ("Stage 1 analysis in UK Biobank" (Results)).

- *If the PBX3 variants are non-coding, what is the proposed effect? Are they splicing, are they in enhancer-like elements? Are they in non-coding RNA-like elements?*

This information was presented in Table S7 ( *Extended data*). We have added text to "Functional annotation" (Results) to provide clarity.

**Is the work clearly and accurately presented and does it cite the current literature?** *Yes*

**Is the study design appropriate and is the work technically sound?** *Yes*

**Are sufficient details of methods and analysis provided to allow replication by others?** *Partly*

**If applicable, is the statistical analysis and its interpretation appropriate?** *Partly*

**Are all the source data underlying the results available to ensure full reproducibility?** *Yes*

**Are the conclusions drawn adequately supported by the results?** *Partly*

# Reviewer report: Reviewer response for version 1

**DOI:** 10.21956/wellcomeopenres.19042.r47012

**Published:** 2021-12-23

**Authors:** Chikashi Terao

**Affiliations:** 1 Laboratory for Statistical and Translational Genetics, RIKEN Center for Integrative Medical Sciences, Yokohama, Japan

The authors conducted a GWAS of hospitalized respiratory infection using UKB and 11 other cohort data. They found PBX3 as a potential susceptibility gene but failed to replicate the findings.

 While they conducted many downstream analyses, most of these analyses (such as fine-mapping, functional annotation, gene expression, and colocalization with eQTL) should have been done for GWAS significant variants. They conducted an assessment of 'sentinel variants' in published GWAS, but this analysis should be done in a meta-analysis, not in a discovery cohort. I don't think that the work is clearly presented since I cannot understand their analyses, such as conditioning analyses focusing on sentinel variants.

 I believe that they first concluded that no GWAS significant signals were identified in the current study and then should move to analyze polygenic architecture, heritability, evaluation of previously reported signals.

Is the work clearly and accurately presented and does it cite the current literature?

Partly

If applicable, is the statistical analysis and its interpretation appropriate?

Partly

Are all the source data underlying the results available to ensure full reproducibility?

Partly

Is the study design appropriate and is the work technically sound?

Partly

Are the conclusions drawn adequately supported by the results?

No

Are sufficient details of methods and analysis provided to allow replication by others?

Partly

Reviewer Expertise:

Genetics

I confirm that I have read this submission and believe that I have an appropriate level of expertise to state that I do not consider it to be of an acceptable scientific standard, for reasons outlined above.

# Response

**Published:** 2023-03-29

**Authors:** Alexander Williams

**Affiliations:** University of Leicester, UK

*The authors conducted a GWAS of hospitalized respiratory infection using UKB and 11 other cohort data. They found PBX3 as a potential susceptibility gene but failed to replicate the findings.*

 We agree that the finding from *PBX3* did not replicate. This involved substantial new analyses across 11 cohorts internationally in Stage 2. In this sense, the Stage 2 analysis successfully achieved its purpose of demonstrating no evidence of replication in independent populations with over 50,000 cases – that is, over 2.5-times the number of cases available in Stage 1 (discovery). Thank you for highlighting that the analyses could have been presented more clearly. We have added a new Figure (Figure 1) to clarify the study design, we have more clearly labelled the analyses "Stage 1" (discovery) and "Stage 2" (follow-up of 40/56 of the top independent sentinel SNPs from Stage 1 in independent populations) and for the 56 sentinels selected for Stage 2, we present in a single table the results from Stage 1 and Stage 2 (for the 40 SNPs available in Stage 2).

*While they conducted many downstream analyses, most of these analyses (such as fine-mapping, functional annotation, gene expression, and colocalization with eQTL) should have been done for GWAS significant variants.*

 We have edited the text at the beginning of the results to convey more clearly the single locus which reached genome-wide significance in Stage 1 (discovery). We accept that interpretation of functional evidence relating to signals which have not reached genome-wide significance need to be interpreted with caution and we have added a statement to the discussion to this effect (end of paragraph 6 in Discussion). We did not exclude these analyses from the paper as, whilst they need to be interpreted with caution pending further evidence on genetic associations from additional populations, they may prove a valuable resource to the community. In addition, analyses such as the pathway analysis used the full genome-wide summary statistics (deposited as part of the downloadable data for this manuscript).

*They conducted an assessment of 'sentinel variants' in published GWAS, but this analysis should be done in a meta-analysis, not in a discovery cohort.*

 The further assessment of sentinel variants in independent populations was wholly new research (new association analyses within each study followed by meta-analysis) and was not based on previous publications. The revised text ("Meta-analysis of Stage 1 and Stage 2" (Results)), new Figure 1, and Table S5 clarifies the study design and conduct.

*I don't think that the work is clearly presented since I cannot understand their analyses, such as conditioning analyses focusing on sentinel variants.*

 All sentinel selection was undertaken in Stage 1 (discovery) and subsequent association testing in Stage 2 was based only on sentinel SNPs – this is now clarified in "Initial signal selection and conditional analyses" (Methods). We clarify that the conditional analyses were for the purposes of identifying secondary signals—additional signals that are close to the primary signals but are conditionally independently associated with the phenotype.

*I believe that they first concluded that no GWAS significant signals were identified in the current study and then should move to analyze polygenic architecture, heritability, evaluation of previously reported signals.*

 Thank you for this suggestion. We have added the SNP heritability estimate (9.48%, 95% CI: 5.80% to 13.16%) in "Stage 1 analysis in UK Biobank" (Results). We have also added a polygenic score analysis. We constructed the polygenic score by applying PRS-CS-auto to the summary statistics from a GWAS of a randomly selected half of the original Stage 1 population as the training dataset. PRS-CS-auto utilises a fully Bayesian approach that automatically learns the hyper-parameters from the training dataset, meaning a validation dataset is not needed. We then tested the association of this polygenic score with our hospitalised respiratory infection phenotype in the other half of the Stage 1 population using a logistic regression model adjusted for age, age ^2^, genotyping array, sex, smoking status and the first 10 principal components of ancestry. We found an 8.1% (95% CI: 5.8%-10.5%; *P*=2.50 *×*10 ^-12^) increase in disease odds per standard deviation unit increase in the polygenic score. While the effect estimate is modest, this a highly significant result which is consistent with polygenicity of the hospitalised respiratory infection phenotype. We compare our findings with previously published findings for related traits in paragraph 2 of the discussion, and discuss possible interpretations of our heritability and polygenic score findings and lack of genome-wide signals in the penultimate paragraph of the Discussion.

***Is the work clearly and accurately presented and does it cite the current literature?*** *Partly.*

***Is the study design appropriate and is the work technically sound?*** *Partly*

***Are sufficient details of methods and analysis provided to allow replication by others?*** *Partly*

***If applicable, is the statistical analysis and its interpretation appropriate?*** *Partly*

***Are all the source data underlying the results available to ensure full reproducibility?*** *Partly*

***Are the conclusions drawn adequately supported by the results?*** *No*

 Thank you for highlighting that the study design and results were not more clearly presented. We hope that the clearer presentation of both design and results provides the transparency we aim for. Based on the reviewer’s suggestions regarding the inclusion of heritability estimates, we now include this finding in our discussion and conclusions, and expand our discussion of the relevance of our findings in the light of lack of evidence of replication from the collectively large independent follow up populations studies in our Stage 2 analyses. In the absence of the corroborative evidence from outside UK Biobank, we caution against over-interpretation of findings from individual loci.
